# Association between sedentary behavior and the risk of dementia: a systematic review and meta-analysis

**DOI:** 10.1038/s41398-020-0799-5

**Published:** 2020-07-06

**Authors:** Shijiao Yan, Wenning Fu, Chao Wang, Jing Mao, Bing Liu, Li Zou, Chuanzhu Lv

**Affiliations:** 1 School of International Education, Hainan Medical University, Haikou, Hainan China; 2 School of Nursing, Tongji Medical College, Huazhong University of Science and Technology, Wuhan, Hubei China; 3 School of Public Health, Tongji Medical College, Huazhong University of Science and Technology, Wuhan, Hubei China; 4 Center of Health Administration and Development Studies, Hubei University of Medicine, Shiyan, Hubei China; 5 Department of Neurology, Taihe Hospital, Hubei University of Medicine, Hubei, China; 6 Department of Emergency, Hainan Clinical Research Center for Acute and Critical Diseases, The Second Affiliated Hospital of Hainan Medical University, Haikou, Hainan China; 7 Emergency and Trauma College, Hainan Medical University, No. 3 Xueyuan Road, Longhua Zone, Haikou, Hainan China; 8 Key Laboratory of Emergency and Trauma of Ministry of Education, Hainan Medical University, Haikou, Hainan China

**Keywords:** Neuroscience, Diseases

## Abstract

An increasing number of original studies suggest that sedentary behavior is associated with the risk of dementia, but the results remain inconsistent and inconclusive. In this meta-analysis, we analyzed available observational epidemiological evidence to identify the association between sedentary behavior and the risk of dementia. We searched PubMed and Embase from their inception to March 2019 to identify observational studies examining the association between sedentary behavior and risk of dementia. Two authors independently extracted data and assessed study quality using predefined criteria. The *Q* statistics and *I²* methods were used to test for heterogeneity. The publication bias of the included studies was also estimated using Begg’s and Egger’s tests. We identified 18 relevant cohort studies involving 250,063 participants and 2269 patients with dementia. Pooled result showed that sedentary behavior was significantly associated with increased risk of dementia (*RR* = 1.30; 95% CI: 1.12–1.51). In addition, subgroup analyses by state, and controlling for the concomitant effects of age, sex, education were conducted for the increase of dementia risk, relating to sedentary, respectively. In general, these subgroup analyses showed no statistically significant differences. The results of our meta-analysis suggested that sedentary behavior was independently associated with a significantly increased risk of dementia, which might have important implications in conducting etiological studies for dementia and developing strategies for dementia prevention.

## Introduction

With the consistent improvement in living standards, human life expectancy has increased. Some studies have shown that the prevalence of dementia is also appears to be gradually increasing^1,2^. Dementia is a complex neurological disorder with an irreversible and nonlinear development process. Dementia does not have only a variety of complex causes, but also will brings with many other health problems. Currently, dementia is the fourth leading cause of death following cancer, heart disease, and cerebrovascular disease in the elderly^3,4^. According to the International Association of Alzheimer’s Disease International Statistics Annual Report 2011, ~36 million people worldwide currently experience dementia, and the incidence is increasing at a rate of 1 patient every 7 s. It is estimated that the number of individuals experiencing senile dementia will increase to 66 million by 2030 and reach 115 million by 2050^5^. Studies have shown that reducing the modifiable risk factors of dementia may contribute to the prevention and control of dementia^6,7^; hence, identifying its possible risk factors is significantly important.

In recent years, there has been a growing interest in physical exercise as a non-pharmacological treatment for dementia^8,9^. Certainly, some studies have reported that regular physical exercise can favorably affect the physical and cognitive functions of patients with dementia^10,11^. Conversely, physical inactivity and a sedentary lifestyle are considered the risk factors of dementia. Studies assessing the association between sedentary behavior and the occurrence of dementia have increased in recent years. However, until now, there have been no consistent conclusions regarding the association between sedentary behavior and the risk of dementia. To the best of our knowledge, the risk of dementia and sedentary lifestyle has not yet been evaluated by a meta-analysis, which is a generally accepted statistical tool for combining results to produce a more precise estimation of associations in different studies^12,13^. Therefore, we conducted a meta-analysis of 18 cohort studies to evaluate the association between sedentary behavior and the risk of dementia. Considering the significant health and economic burden of dementia, the results of our study may provide additional practical and valuable treatments for dementia prevention.

## Materials and methods

Approval by institutional review boards is not required for this systematic review of previously published de-identified data.

### Literature search strategy

We conducted this meta-analysis in accordance with the Preferred Reporting Items for Systematic Reviews and Meta-Analyses^14^ and the checklist of items in the Meta-Analysis Of Observational Studies in Epidemiology^15^. We performed a comprehensive search on PubMed, Embase, and Web of Science databases from their inception to March 2018 for cohort studies published in peer-reviewed journals describing an association between sedentary behavior and the risk of dementia. We used the following keywords to identify relevant citations: “sedentary” or “sedentariness” or “long time sitting” in combination with “dementia” or “Alzheimer’s disease” or “AD” or “cogniti”. Only articles published in the English language were considered. In addition, reference lists of the retrieved original articles and relevant review articles were also comprehensively examined to identify further pertinent studies.

### Study selection

Studies meeting the following criteria were included in the meta-analysis: (1) the study design was cohort; (2) sedentary behavior was the exposure variable and the outcome was the incidence of dementia; (3) the study reported the relative risks (RRs) with the corresponding 95% confidence intervals (CIs) of dementia associated with sedentary behavior or included the data needed for their calculation. Animal studies, clinical trials, reviews, letters, and commentaries were excluded. Only studies with detailed information on both sedentary behavior and the incidence of dementia were included.

### Data extraction and quality assessment

Two authors (S.Y. and W.F.) independently extracted data in a standardized fashion. The following data were included: first author, publication year, country (state), age, sample size, cases, adjusted factors, and adjusted RR with 95% CI. Discrepancies were resolved by a discussion with a third author (L.Z.). Adjusted RRs were selected over the unadjusted risk estimates. In cases where multiple risk estimates were reported in the same study, for example, when the study introduced risk estimates for 3 different years, those risk estimates were included as separate risk estimates.

Quality assessment was performed according to the Newcastle-Ottawa quality assessment scale^16^, which is a validated scale for nonrandomized studies in meta-analyses. This scale assigns a maximum of nine points to each study: four for the selection of participants and measurement of exposure, two for comparability of cohorts or cases and controls based on the design or analysis, and three for the assessment of outcomes and adequacy of follow-up. We assigned scores of 0–3, 3.5–6, and 6.5–9 for low-, moderate-, and high-quality studies, respectively. When the studies had several adjustment models, we extracted those that reflected the maximum extent of adjustment for potentially confounding variables. Disagreement between the two authors’ independent data analysis was resolved through a review by the third author.

### Statistical analyses

The *RR* was considered as the common measure of the association between sedentary behavior and the risk of dementia. When *RRs* were reported separately for subgroups by the different sedentary times in one study, the fixed-effects model was used to combine the results of the subgroups and calculate a common *RR* for the main analysis^12^. In addition, the random-effects model was used to calculate an overall pooled *RR* for the main analysis.

Heterogeneity was identified according to Cochran’s *Q* test, with *P* < 0.10 indicating heterogeneity. The *I*^2^ statistics measured the percentage of total variation through studies due to heterogeneity rather than chance. It was calculated according to the formula by Higgins^17^. *I*^*2*^ was used to quantify the heterogeneity, with 25%, 50%, and 75% indicating low, moderate, and high degrees of heterogeneity, respectively.

Subgroup analyses were performed to determine the possible influence of factors such as state or region and weather. The concomitant effects of age, sex, and educational level were controlled in some studies. The Begg’s rank correlation and the Egger’s linear regression tests were used to assess potential publication bias^18,19^. Moreover, the Duval and Tweedie’s nonparametric trim-and-fill method were used to adjust potential publication bias^20^. All analyses were performed using the Stata statistical software (version 12.0; College Station, TX, USA), and all tests were two-sided with a significance level of 0.05.

## Results

### Literature search

**Fig. 1:**
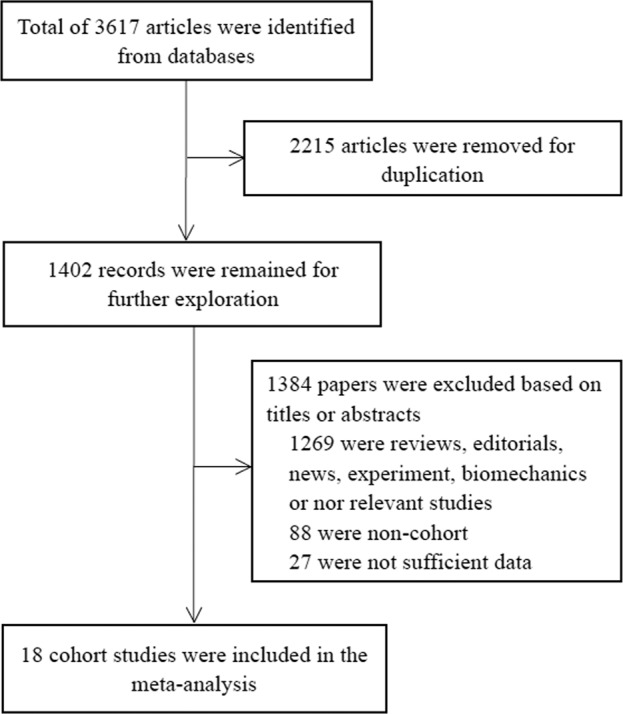
Flow chart of study identification.

A total of 3617 articles were searched from the electronic databases, and 2215 articles were removed because of duplication. Another 1402 unqualified records were excluded after browsing the titles or abstracts. Among these, 1269 papers reported irrelevant studies, which did not analyze the association between sedentary behavior and the risk of developing dementia, 88 were non-cohort studies, and 27 articles offered insufficient information. After retrieving and reviewing the full text, we determined that 18 cohort studies met our inclusion criteria^21–38^. The study selection procedure is presented in the flow chart in Fig. 1.

### Characteristics of the included studies

Table 1 shows the main characteristics of the 18 cohort studies included in the systematic review. The 18 cohort studies were published between 1984 and 2018. Most of these studies (11) were conducted in Europe (*RR* = 1.37, 95% CI = 1.14–1.65), four in America (*RR* = 1.52, 95% CI = 1.30–1.77), two in Asia (*RR* = 0.76, 95% CI = 0.52–1.11), and one in Australia (*RR* = 0.98, 95% CI = 0.89–1.08). The sample size of these studies ranged from 33 to 490 with a total of 250,063 participants. Seventeen studies were published after 2000, and only one study was published before 2000. The quality assessment scores ranged from 6 to 9, with an average score of 7 points, representing satisfactory quality of the studies.

**Table 1:** Main characteristics of the included studies involving sedentary behavior and the risk of dementia.

Author	Year	Country (state)	Age	Sample size	Cases	Adjustments
Deckers K.	2017	UK (Europe)	87.7 ± 2.7	323	76	Age, sex and educational level
Kishimoto H.	2016	Japan (Asia)	≥65	803	93	NR
T. Luck	2016	Germany (Europe)	NR	217,000	NR	NR
MacDonald J.P.	2015	Canada (America)	≥50	5219	NR	NR
T. Luck	2014	Germany (Europe)	81.1 ± 3.5	2492	278	Age, gender, level of education, MMSE scores, comorbidity
Mehlig K.	2014	Sweden (Europe)	38-60	1448	165	Baseline covariates age, education, smoking, consumption of alcohol, triglycerides, hypertension, and parental history of diabetes
Norton S.	2014	Sweden (Europe)	82.1 ± 5	180	36	Education, portion of fruits/vegetables in diet, current smoking status, alcohol consumption, body-mass index, and angina pectoris
de Bruijn R.F.	2013	Netherlands (Australia)	72.7 ± 7.2	4406	490	Age, sex, score on MMSE, low educational level, smoking, APOE-e4 carrier status, hypertension, BMI, diabetes, total cholesterol, and HDL-cholesterol
Verdelho A.	2012	Portugal (Europe)	65-84	639	34	Age, educational level, sex, white matter changes severity, and medial temporal atrophy
Gelber R.P.	2012	American	71–93	3468	117	Age, years of education, APOE e4 status, childhood years spent in Japan, occupational status, high cholesterol, and history of hypertension, diabetes, and cardiovascular disease
Keller L.	2010	Sweden (Europe)	NR	936	243	Age, gender, education and APOE, TT, BMI, diabetes, CVD
Scarmeas N.	2009	American	77	1880	282	Age, sex, ethnicity, education, apolipoprotein E ε4 allele, caloric intake, body-mass index, smoking, depression, leisure activities, comorbidity index, baseline Clinical Dementia Rating score, and time between first dietary and first physical activity assessment
Kivipelto M.	2008	Finland (Europe)	70.8 ± 3.7	1284	57	Age, sex, follow-up time, education, body-mass index, serum cholesterol, systolic blood pressure, myocardial infarction, stroke, diabetes mellitus and ApoE4 carriers status sex, education, follow-up time, locomotor disorders, APOE 4 genotype, midlife body-mass index, systolic blood pressure, cholesterol, and history of myocardial infarction, stroke, diabetes mellitus, smoking status, and alcohol drinking
Rovio S.	2007	Finland (Europe)	70	1158	33	NR
Rovio S.	2005	Finland (Europe)	70.9 ± 3.9	1935	76	Age at re-examination, APOE 4 genotype, midlife body-mass index, systolic blood pressure, cholesterol, and history of myocardial infarction, stroke, diabetes mellitus, smoking status and alcohol drinking
Anttila T.	2003	Finland (Europe)	74 ± 4	1449	70	Age and APOE ε4 carrier status
Laurin D.	2001	Canada	80	4615	169	Age, sex, and educational level
Yoshitake T.	1995	Japan (Asia)	Men: 73 ± 5.6 Women: 74 ± 6.1	828	50	Age

### Results of meta-analysis

#### Association between sedentary behavior and the risk of dementia

**Fig. 2:**
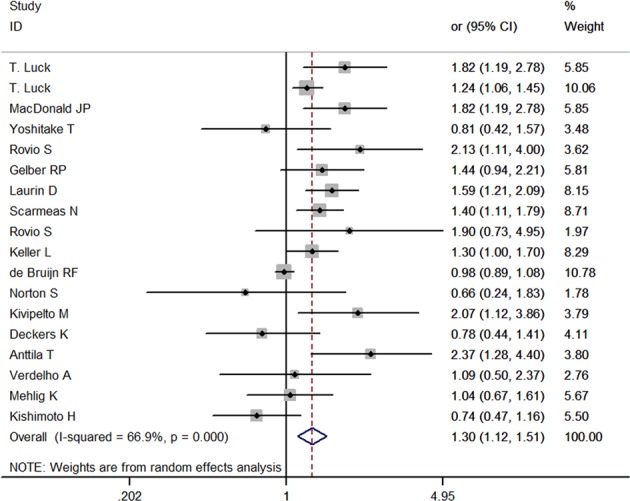
Forest plot of sedentary behavior associated with dementia.

Figure 2 shows the results from the random-effects model combining the *RR*s for dementia in relation to sedentary behavior. Eighteen studies investigated the association between sedentary behavior and risk of dementia. The pooled *RR* of dementia for sedentary behavior was 1.30 (95% CI, 1.12–1.51), with a substantial heterogeneity across studies (*P* = 0.000, *I*^*2*^ = 66.9%).

### Results of subgroup and sensitivity analyses

#### Subgroup analyses

Table 2 shows the results of subgroup analyses. To assess the stability of the primary estimates outcomes and identify the potential resources of heterogeneity, we performed subgroup analyses. Subgroup analyses by state, controlling for the concomitant effects of age, sex, and educational level, were performed to assess the increased risk of dementia as a result of a sedentary behavior. In general, these subgroup analyses showed no statistically significant difference since the pooled results of all subgroups showed a positive and statistically significant association between sedentary behavior and the risk of dementia.

**Table 2:** Results of subgroup analyses about sedentary behavior and the risk of dementia.

Subgroup	Number of studies	*RR*	95% confidence intervals	*P* for heterogeneity	*I*^2^ (%)	*P* Value for interaction
State						
Asia	2	0.76	0.52–1.11	0.825	0.00	>0.05
America	4	1.51	1.30–1.77	0.733	0.00	
Europe	11	1.37	1.14–1.65	0.066	42.5	
Australia	1	0.98	0.89–1.09	–	–	
Controlling for age						
Yes	5	1.30	0.82–2.06	0.011	69.3	>0.05
No	13	1.29	1.10–1.51	0.000	67.2	
Controlling for sex						
Yes	9	1.29	1.08–1.54	0.000	72.3	>0.05
No	9	1.30	0.98–1.74	0.011	59.8	
Controlling for education						
Yes	12	1.26	1.08–1.47	0.001	64.8	>0.05
No	6	1.42	0.95–2.13	0.006	69.5	

### Sensitivity analyses

We performed sensitivity analyses to determine the potential sources of heterogeneity in the association between sedentary behavior and risk of dementia, examine the influence of various exclusions on the combined *RR*, and assess the robustness of all results. We compared the fixed-effect and random-effect models, but found no significant difference in the pooled RRs between the two (fixed-effects model pooled *RR* = 1.16 [95% CI, 1.09–1.24], random-effects model pooled *RR* = 1.30 [95% CI, 1.12–1.51]). In addition, each study was excluded in turn, and the results of the remaining studies were pooled together. The pooled RR did not significantly change, ranging from 1.27 (95% CI, 1.10–1.47) to 1.34 (95% CI, 1.16–1.56), and the heterogeneity was detected with an *I*^2^ = 65.4% and 66.3%, respectively.

### Publication bias

**Fig. 3:**
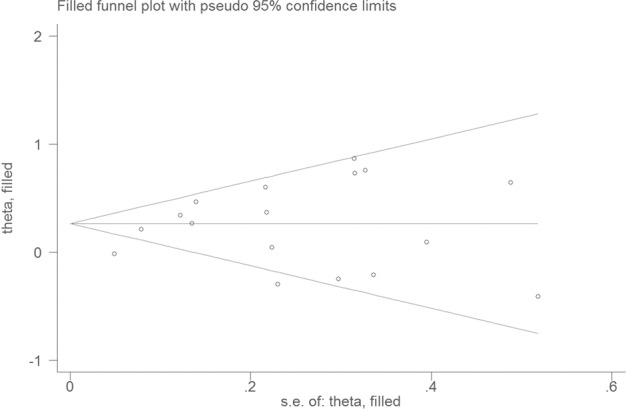
Funnel plot for studies of sedentary behavior and dementia. The horizontal line represents summary effect estimates, and two slashes lines are pseudo 95% CIs.

**Fig. 4:**
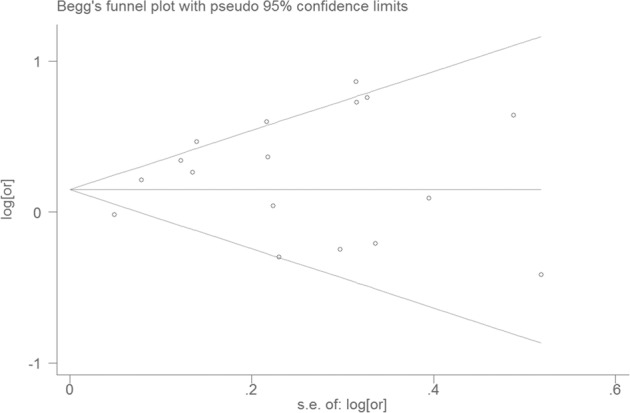
Filled funnel plot of *RR* from studies that investigated the association between sedentary behavior and the risk of dementia. The horizontal line represents summary effect estimates, and two slashes lines are pseudo 95% CIs.

Visual inspection of the funnel plot showed significant asymmetry (Fig. 3). The Egger and Begg test indicated no evidence of publication bias (Egger, *P* = 0.677; Begg, *P* = 0.074). The trim-and-fill method was used to evaluate the impact of any potential publication bias, and the results showed that two potentially missing studies would be required to obtain the funnel plot symmetry for the association between sedentary behavior and the risk of dementia (Fig. 4). After using the trim-and-fill method, the corrected *RR* was 1.27 (95% CI, 1.10–.47; random-effects model, *P* = 0.000), suggesting that the pooled *RR* did not significantly change by the correction for potential publication bias.

## Discussion

With the rapid development of information technology, people are able to receive essential information without leaving their homes. Artificial intelligence and mobile terminals (such as mobile phones and laptops) enable individuals to interact with other individuals worldwide in a static or sedentary manner. Therefore, sedentary lifestyle has been considered to be significantly prevalent in the current society, specifically for office workers, students, and drivers. Previous reviews have suggested that sedentary behavior was associated with lower cognitive performance, although the attributable risk of sedentary time to all-cause dementia incidence is unclear^39,40^. However, the meta-analysis of 18 cohort studies involving 250,063 participants and 2269 patients confirmed the positive association between sedentary behavior and the risk of dementia. Compared with individuals who had not been exposed to sedentary behavior, individuals who were exposed to sedentary behavior had a 30% higher risk of experiencing dementia. Sedentary behavior was associated with several chronic diseases that were also associated with cognitive impairment and risk of dementia^41,42^. Previous data suggested that prolonged sedentary time could impair glucose and lipid metabolism^43–45^, which were recognized as the risk factors for cognitive decline and all-cause dementia^46,47^. In addition, inflammation was also identified as a potential risk factor for dementia^48,49^. Meanwhile, sedentary behavior might conversely induce or aggravate individual inflammation^50–52^. Finally, exercise was considered a protective factor for dementia^53^. However, physical activity declines with age, while sedentary behavior increases with age^54,55^. Voss and his colleagues found that increases in sedentary behavior and sedentary time were significantly observed in individuals who were about to retire and were continuously observed in adults after the age of 60 years, and individuals aged over 80 years are often involved in sedentary behavior, with an average sedentary time of 9 h per day^56^, eliminating the benefits of physical exercises on individual’s cognitive health.

Considering that a substantial heterogeneity was observed in the included studies, we further performed a subgroup analysis to determine the potential sources of heterogeneity. Based on the result of the subgroup analyses, we found that the pooled odds ratios of the studies conducted in Asia and Australia showed no statistically significant association between sedentary behavior and the risk of dementia. The probable explanation was that only two studies from Asia and one from Australia were included in this meta-analysis. Their sample sizes were small and lacked representation, thereby potentially leading to bias in the results^57^, indicating the need for additional studies in Asia and Australia to determine significant robust results. When the analysis was stratified to control the concomitant effects of age, sex, and educational level, we found that the summarized results from the original studies without adjusting for age, sex, and educational level showed no statistically significant difference in the association between sedentary behavior and risk of dementia (*P* > 0.05). On the contrary, if the original studies were controlled for age, sex, and educational level, the merged *RR*s were all statistically significant (*P* < 0.05), although the difference between the pooled results in each subgroup was not statistically significant (*P* > 0.05). Specifically, aging was an independent suggested risk factor for developing dementia^58,59^. Our subgroup analysis suggested that age might be a confounding factor in the association between sedentary lifestyle and risk of dementia, consistent with the results when sex and educational level were adjusted. To generalize the findings, more studies determining the association between age, sex, and educational level and sedentary lifestyle and their synergistic effects on the risk of dementia were required.

To assess the consistency and robustness of the results from the primary analysis and determine the potential sources of heterogeneity in the association between sedentary behavior and the risk of dementia, we performed a sensitivity analysis. However, the pooled results showed only minor changes ranging from 1.27 (95% CI, 1.10–1.47) to 1.34 (95% CI, 1.16–1.56) after excluding each study in turn from the analysis, highlighting the robustness and reliability of the primary result. On the contrary, publication bias was observed in the included studies, but the primary result did not significantly change after using the “trim-and-fill” method, significantly confirming the robustness of our findings.

This meta-analysis has several strengths. First, the study design of all the studies in our analysis was cohort, which was considered as a stronger measure when demonstrating causation and identifying risk factors than other observational study designs^60^. Second, consistent results from the sensitivity analysis indicated that our findings were reliable and robust, although heterogeneity was observed among the studies. Third, when several *RRs* were introduced separately in terms of the different sedentary times, we combined the results of these subgroups and calculated a common result using the fixed-effects model. Therefore, we could pool the outcomes regarding the association between the risk of dementia and sedentary behavior with non-sedentary behavior.

On the contrary, this meta-analysis has several limitations that may affect the interpretation of the results in our analysis. First, the heterogeneity of the included studies was significant (*I*^*2*^ > 60%) and was observed throughout the analyses. However, we determined the potential sources of heterogeneity by performing subgroup analyses and sensitivity analysis. Second, considering the limited information provided in original studies, a dose–response analysis was not performed to provide further evidence supporting the association between sedentary behavior and the risk of dementia. Third, the definition of “sedentary behavior” was inconsistent, which might induce bias in effect size estimates. Fourth, the outcome we were interested in was all-cause dementia. Considering that the information related to the subtypes of dementia was insufficient, we did not analyze the effects of sedentary lifestyle on specific dementia subtypes, such as vascular dementia and senile dementia.

## Conclusion

The results of this meta-analysis of cohort studies with the most up-to-date evidence suggested that sedentary behavior was significantly associated with an increased risk of dementia. The positive association remained consistent throughout the analysis. Considering the increasing prevalence of sedentary behavior in modern society, our findings have been significant for policy makers and health education institutions. Considering the insufficient information on the dose–response association of sedentary time and the risk of developing dementia, well-designed studies with adequate analyses of sedentary time and the risk of dementia are required to confirm our findings.
