# Correction: Association between sedentary behavior and the risk of dementia: a systematic review and meta-analysis

**DOI:** 10.1038/s41398-020-00891-6

**Published:** 2020-07-06

**Authors:** Shijiao Yan, Wenning Fu, Chao Wang, Jing Mao, Bing Liu, Li Zou, Chuanzhu Lv

**Affiliations:** 1 School of International Education, Hainan Medical University, Haikou, Hainan China; 2 School of Nursing, Tongji Medical College, Huazhong University of Science and Technology, Wuhan, Hubei China; 3 School of Public Health, Tongji Medical College, Huazhong University of Science and Technology, Wuhan, Hubei China; 4 Center of Health Administration and Development Studies, Hubei University of Medicine, Shiyan, Hubei China; 5 Department of Neurology, Taihe Hospital, Hubei University of Medicine, Hubei, China; 6 Department of Emergency, Hainan Clinical Research Center for Acute and Critical Diseases, The Second Affiliated Hospital of Hainan Medical University, Haikou, Hainan China; 7 Emergency and Trauma College, Hainan Medical University, No. 3 Xueyuan Road, Longhua Zone, Haikou, Hainan China; 8 Key Laboratory of Emergency and Trauma of Ministry of Education, Hainan Medical University, Haikou, Hainan China

Correction to: *Translational Psychiatry*

10.1038/s41398-020-0799-5 published online 21 April 2020

In the original Article, the authorship contribution statement named four authors instead of two. “These authors contributed equally: Shijiao Yan, Wenning Fu, Li Zou, Chuanzhu Lv” should read “These authors contributed equally: Shijiao Yan, Wenning Fu”.

This has now been corrected in both the PDF and HTML versions of the Article.

